# Medical Education in America

**Published:** 1897-07-17

**Authors:** 


### MEDICAL EDUCATION IN AMERICA.

In a presidential address delivered before the American Academy of Medicine, Dr. J. C. Wilson spoke of the progressive lengthening of the students' curriculum which had taken place all over the States. Instead of two annual sessions of scarcely five months each, the student had now to attend four annual sessions of seven months or even more.

The standard of the entrance examinations also appears to be gradually rising not in one State alone but all over the country, and what is very important is that many of the colleges have relinquished their charter rights according to which their diplomas conferred the right to practice. This movement in favour of State examination was, no doubt, aimed primarily against the quacks and the diploma mills which flourished so exceedingly in many of the States, but in its more recent developments its effect has been to deal serious blows at the inadequately equipped colleges, legally chartered though they might be.

